# Giant testicular seminoma with atypical painful presentation and delayed diagnosis: a case report

**DOI:** 10.1093/jscr/rjag342

**Published:** 2026-04-30

**Authors:** Fuad Haj Ali, Eyad Jamileh, Faeza Limbada

**Affiliations:** Department of Urology, Royal Blackburn Hospital, East Lancashire Hospitals NHS Trust, Haslingden Road, Blackburn BB2 3HH, England, United Kingdom; Department of Urology, Royal Blackburn Hospital, East Lancashire Hospitals NHS Trust, Haslingden Road, Blackburn BB2 3HH, England, United Kingdom; School of Medicine, Queens University Belfast, University Road, Belfast BT7 1NN, Northern Ireland, United Kingdom; Department of Urology, Royal Blackburn Hospital, East Lancashire Hospitals NHS Trust, Haslingden Road, Blackburn BB2 3HH, England, United Kingdom

**Keywords:** seminoma, testicular tumour, case report, testicular mass, cancer

## Abstract

Giant testicular tumours are rare in contemporary practice and are typically associated with delayed diagnosis and advanced disease. We report a 32-year-old male presenting with a 1-month history of progressively enlarging, painful right-sided scrotal swelling, initially mischaracterized on ultrasound imaging performed abroad. Examination revealed marked scrotal enlargement with overlying erythema and a buried penis. Computed tomography demonstrated a large right testicular mass with retroperitoneal lymphadenopathy, without distant metastases. Serum tumour markers showed elevated β-human chorionic gonadotropin and lactate dehydrogenase, with normal alpha-fetoprotein. The patient underwent urgent radical inguinal orchidectomy. Histopathology confirmed a pure seminoma measuring 15.8 cm with lymphovascular invasion and spermatic cord involvement (pT3). Post-operatively, tumour markers declined significantly, and the patient was commenced on bleomycin, etoposide, and cisplatin chemotherapy. This case emphasizes that painful scrotal swelling does not exclude malignancy and highlights the importance of early recognition and multidisciplinary management.

## Introduction

Testicular cancer is the most common solid malignancy in young adult men, with peak incidence between 20 and 40 years [1]. Most cases arise from germ cells and are classified into seminomatous and non-seminomatous tumours, with seminomas accounting for approximately half of diagnoses [2]. Clinically, it typically presents as a painless testicular mass; however, some patients may present with pain or swelling, which can lead to misdiagnosis and delayed treatment [1, 3]. Diagnosis is based on scrotal ultrasonography, serum tumour markers, and staging imaging [3]. Although prognosis is excellent with early detection, delayed diagnosis may result in advanced disease requiring multimodal treatment [4]. This case describes an atypical presentation of a large seminoma with rapid progression and associated pain.

## Case presentation

A 32-year-old male presented to a district general hospital with a 1-month history of progressively enlarging, painful right-sided scrotal swelling. Before presentation, he had been evaluated abroad, where a scrotal ultrasound was reportedly reassuring. He denied lower urinary tract symptoms, trauma, or systemic features such as fever or weight loss. He had no past medical history, no known risk factors for testicular malignancy, and no relevant family history.

**Figure 1:**
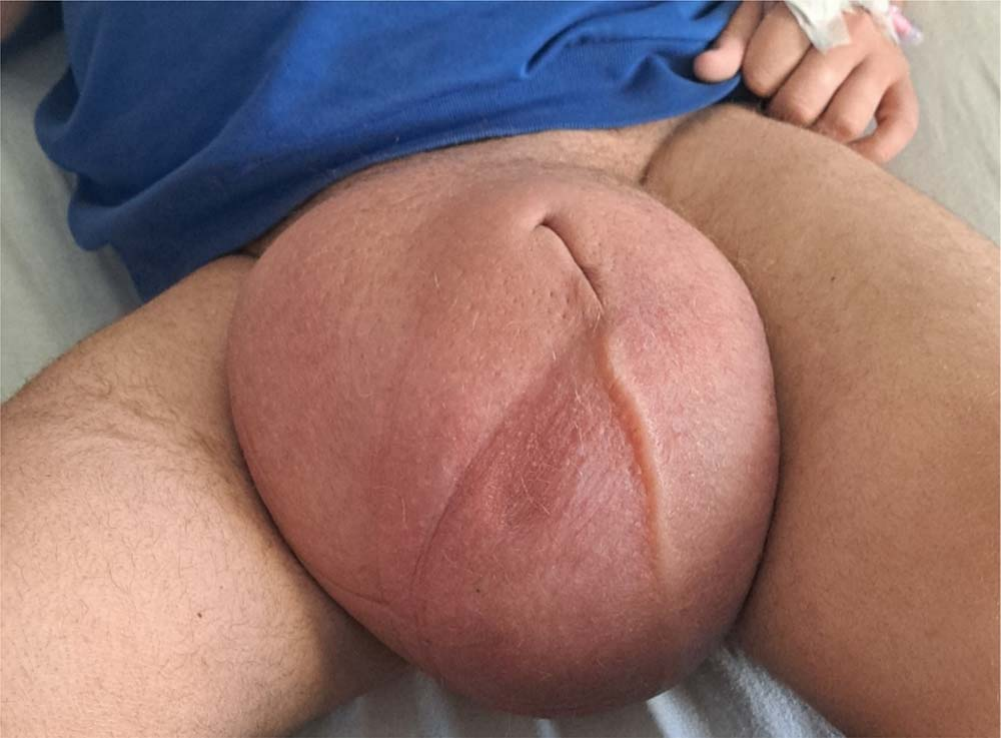
Gross appearance of the scrotum demonstrating marked enlargement of the right hemiscrotum with overlying skin erythema and buried penis due to underlying testicular mass.

On physical examination, there was a markedly enlarged scrotum measuring ~40 cm by 25 cm, with associated oedema and erythema of the overlying skin. The penis was buried within the swelling (Fig. 1). Prehn’s sign was positive, and there was no cough impulse. There was no lymphadenopathy, and abdominal examination was unremarkable.

**Figure 2:**
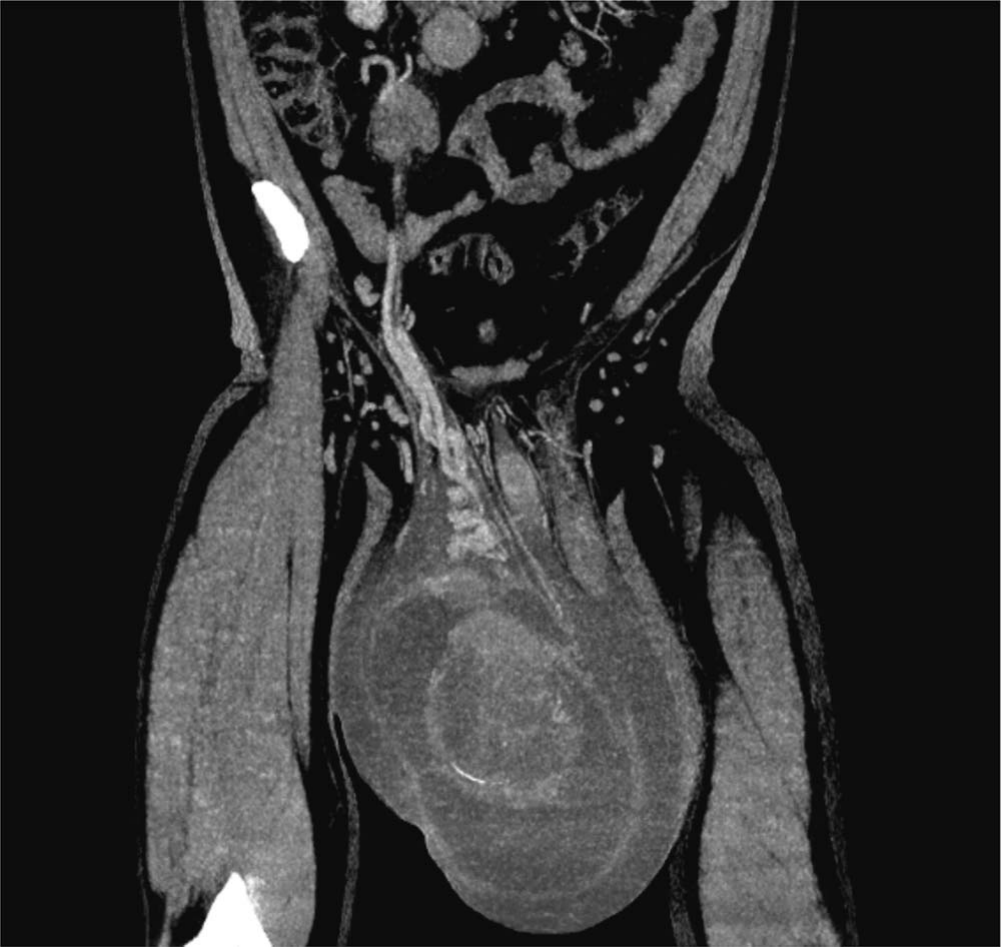
Contrast-enhanced coronal CT image demonstrating a large heterogeneous right testicular mass occupying the hemiscrotum, with associated hydrocele. The mass extends superiorly along the course of the right testicular vessels, with multiple enlarged retroperitoneal lymph nodes in the para-aortic and precaval regions, consistent witic spread.

A contrast-enhanced computed tomography (CT) scan of the chest, abdomen, and pelvis demonstrated a large right testicular mass with associated retroperitoneal lymphadenopathy, with the largest node measuring 3 cm in short-axis diameter. There was no evidence of thoracic or intracranial metastases (Fig. 2).

Serum tumour markers demonstrated an elevated β-human chorionic gonadotropin (β-hCG) level of 131 IU/L and lactate dehydrogenase (LDH) of 1084 U/L, with a normal alpha-fetoprotein (AFP). In the context of the clinical and radiological findings, a provisional diagnosis of a primary testicular malignancy was made.

**Figure 3:**
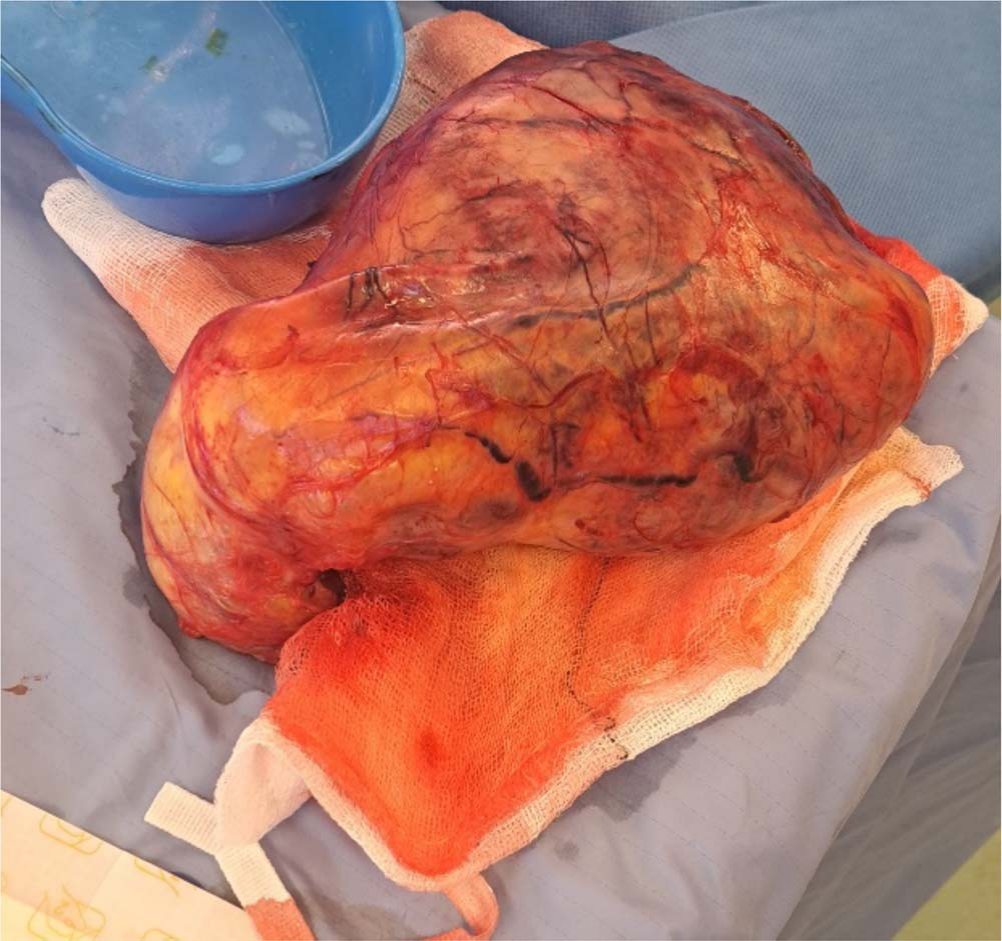
Intraoperative photograph of the excised specimen following right radical orchidectomy, demonstrating a markedly enlarged testicular tumour with a smooth, tense external surface and prominent superficial vascularity.

Following multidisciplinary team discussion, the patient underwent urgent radical inguinal orchidectomy. Due to the significant size of the mass, an extended inguinoscrotal incision was required to facilitate adequate exposure. The spermatic cord was idenssected, and secured at a high level. A hydrocele was encountered and drained intraoperatively. The testis was noted to be markedly enlarged and was dissected free from the surrounding scrotal tissues and delivered en bloc. Haemostasis was achieved, and the wound was closed in layers. An intraoperative image of the excised specimen is shown in Fig. 3.

The patient’s immediate postoperative course was uncomplicated. He was mobilized early and discharged following an uneventful recovery. Post-operatively, tumour markers showed a significant decline, with β-hCG decreasing to 6 IU/L and LDH to 337 U/L, whilst AFP remained within normal limits. This is summarized in Table 1.

**Table 1:** Pre-operative and post-operative serum tumour markers.

Tumour Marker	Pre-operative	Post-operative
AFP	Within normal limits	Within normal limits
β-hCG (IU/L)	131	6
LDH (U/L)	1084	337

Abbreviations: AFP – alpha-fetoprotein; β-hCG – beta-human chorionic gonadotropin; LDH – lactate dehydrogenase.

Histopathological examination confirmed a pure seminoma measuring 15.8 cm in maximum dimension. Macroscopically, the tumour demonstrated a heterogeneous cut surface with areas of haemorrhage and necrosis. Immunohistochemistry showed tumour cells positive for OCT3/4, CD117, and placental alkaline phosphatase (PLAP), and negative for AFP, CD30, and AE1/AE3, supporting the diagnosis. There was evidence of lymphovascular invasion, with tumour extension into the hilar soft tissue, tunica vaginalis, and spermatic cord. Surgical resection margins, including the spermatic cord margin, were free of malignancy. Intratubular germ cell neoplasia could not be definitively assessed due to the absence of background testicular parenchyma. Overall, the tumour was staged as pT3.

Following surgical management, the patient was referred to oncology services and commenced on bleomycin, etoposide, and cisplatin chemotherapy.

## Discussion

Giant testicular tumours are uncommon in contemporary clinical practice, largely due to earlier detection and improved awareness [5]. A limited number of cases have reported markedly enlarged testicular malignancies, many presenting with advanced disease and metastatic involvement at diagnosis. Tumours exceeding 20 cm are often associated with pulmonary or retroperitoneal metastases, requiring multimodal treatment including chemotherapy and delayed surgical intervention [5–7]. In contrast, the present case highlights a significantly enlarged seminoma with regional nodal disease but without distant metastasis, allowing for timely surgical management followed by systemic therapy.

Delayed presentation remains key in the development of such large tumours. Misinterpretation of initial investigations and lack of symptom recognition have been implicated. In this case, an initially reassuring ultrasound contributed to a delay in diagnosis despite progressive symptoms, emphasizing the importance of correlating imaging findings with clinical progression and maintaining a low threshold for repeat assessment or specialist referral.

Although testicular cancer classically presents as a painless mass, painful swelling is not uncommon and may lead to diagnostic confusion with benign conditions such as epididymo-orchitis. Clinicians should remain vigilant when assessing young men with scrotal symptoms, particularly when there is rapid progression or failure to respond to initial management.

The radiological findings demonstrated retroperitoneal lymphadenopathy along the expected lymphatic drainage pathway of the testis, including para-aortic and precaval regions. This pattern is characteristic of testicular malignancy and provides an important diagnostic clue.

Radical inguinal orchidectomy remains the cornerstone of management, serving both diagnostic and therapeutic roles [3]. The inguinal approach avoids disruption of lymphatic drainage and potential tumour seeding. In this case, the large tumour required an extended approach, but oncological principles were maintained, including high ligation of the spermatic cord and en bloc resection.

Histopathology confirmed a pure seminoma with lymphovascular invasion and spermatic cord involvement (pT3). Despite these adverse features, seminomas are highly sensitive to platinum-based chemotherapy. The reduction in tumour markers following surgery reflects an appropriate response, and ongoing Bleomycin, Etoposide and Cisplatin (BEP) chemotherapy aligns with current guidelines.

## Conclusion

Giant testicular tumours represent a rare but important presentation of malignancy, most often associated with delayed diagnosis. This case highlights the need for increased awareness, early escalation when findings are discordant, and adherence to oncological principles.
